# Authors’ reply

**Published:** 2010

**Authors:** Vishali Gupta, Amod Gupta, Pawan Gupta

**Affiliations:** Advanced Eye Centre, Department of Ophthalmology, Post Graduate Institute of Medical Education and Research, Chandigarh, India

Dear Editor,

This case report is not about the diagnosis of idiopathic central serous chorioretinopathy (ICSC) at the time of presentation as that is really not the objective of our case report.[1,2] The report is about the optical coherence tomography (OCT) showing changes of acute ICSC before the development of expanding dot sign on fluorescein angiogram. As mentioned in the letter, the fluorescein angiogram in the right eye at presentation is suggestive of forme fruste of ICSC or chronic ICSC and, by no stretch of imagination, appears like a precursor to the development of acute ICSC.

Regarding the question of the inability of raster line scan to pick up all the pigment epithelium detachment (PEDs) seen on the map, we would like to mention the very basic fact that the raster line scan shows the morphological alterations seen at one particular line scan through which the slice navigator is passing. The changes that are seen on the 3D retinal pigment epithelium (RPE) map are mapping the entire area of the cube and thus all the changes seen on the map cannot be seen in a single line scan.[3] None of the other conditions mentioned in the letter, including ICSC, can be diagnosed based on OCT alone and thus this query has no relevance.[4] The OCT scan in this patient showed changes consistent with acute ICSC even before the development of expanding dot sign that is required to make the diagnosis of acute ICSC and thus it definitely scores over fluorescein angiography. Regarding the statement, “We wish to mention that OCT cannot predate any pathognomonic changes in CSCR,” it would be interesting to see the reference to this statement.

## References

[1] Barange KK, Saha D (2010). Comment on: Spectral domain optical coherence tomography predates fluorescein angiography in diagnosing central serous chorioretinopathy. Indian J Ophthalmol.

[2] Gupta V, Gupta A, Gupta P (2010). Spectral domain optical coherence tomography predates fluorescein angiography in diagnosing central serous chorio retinopathy. Indian J Ophthalmol.

[3] Gupta P, Gupta V, Dogra MR, Singh R, Gupta A (2010). Morphological changes in the retinal pigment epithelium on spectral-domain OCT in the unaffected eyes with idiopathic central serous chorioretinopathy. Int Ophthalmol.

[4] Yannuzzi LA (2010). Central serous chorioretinopathy: A personal perspective. Am J Ophthalmol.

